# Has the High-Tech Industry along the Belt and Road in China Achieved Green Growth with Technological Innovation Efficiency and Environmental Sustainability?

**DOI:** 10.3390/ijerph16173117

**Published:** 2019-08-27

**Authors:** Chang Li, Mingyang Li, Lu Zhang, Tingyi Li, Hanzhen Ouyang, Sanggyun Na

**Affiliations:** 1 School of Business Administration, Wonkwang University, 460 Iksandae-ro, Iksan 54538, Korea; 2 Department of Office Management, Hebei GEO University, Shijiazhuang 050031, China; 3 College of Design, East China Normal University, No.3663, North Zhongshan Road, Shanghai 200062, China

**Keywords:** technological innovation efficiency, high-tech industry, green growth, influence, the Belt and Road

## Abstract

From the perspective of green growth, which seeks to coordinate and make sustainable the development of resources, the environment, and the economy, this study’s aim was to find out whether the high-tech industry along the Belt and Road (B&R) is sustainable and effective in using resources, reducing environmental pollution, and increasing performance. This study used panel data covering 16 provinces (municipalities) along the B&R in China between 2009 and 2016. This study used the directional distance function (DDF) and the global Malmquist–Luenberger (GML) index model to analyze the technological innovation efficiency (TIE) of the high-tech industry (HTI) while considering the undesirable output (environmental pollution). Further, supplemented by ArcGIS geographical analysis, this study carried out a comparative analysis of the TIE and its decomposition in the HTI along the B&R from geographical and time-series dimensions. Moreover, the panel Tobit regression model was used to analyze the influencing factors of TIE. The results show that the direct financial support of the government has no impact on the improvement of TIE in the HTI, the government’s regulation of environmental pollution can significantly affect the improvement of the TIE, the intensity of R&D has a significantly negative impact on the TIE, a higher level of R&D personnel in the HTI can be helpful in improving TIE, and increasing the import and export trade volumes of the HTI can promote TIE.

## 1. Introduction

Severe environmental pollution and increasing resource constraints make the reduction of pollutant emission and the sustainable utilization of resources a global issue [1,2]. In the past, in order to pursue rapid economic growth, the emphasis on wasting resources and abandoning environmental standards often seems to have ignored the importance of sustainable development [3]. By noticing the importance of sustainable development, the debate on how to balance socio-economic development with resource constraints has largely been discussed. However, recent discussion has increasingly shifted to the concept of green growth with reports by influential international organizations [4,5,6]) dedicated to this issue. Green growth seeks to coordinate long-term sustainability with short-term benefits to achieve both environmental sustainability and economic growth [3]. Technical innovation is key to achieve green growth by creating more efficiency in the utilization of resources, reducing waste and energy consumption, and creating the highest value [7,8]. More countries nowadays look to ensure sustainable economic growth and industrial technological innovation development within an environmentally viable framework to achieve green growth [9,10].

China is also committed to achieving green growth, with emphasis on technological innovation. Facing a structural imbalance that cannot be ignored, which is characterized by a high proportion of low added value, high consumption, high pollution, and high emission industries and a low proportion of high added value, international competitiveness, and green industries, the Chinese economy is committed to changing from the extensive growth driven by capital and labor factors to intensive growth that relies on technological progress and innovation, without ignoring environmental pollution [11]. In 2015, the National Committee officially announced the “Made in China 2025” campaign, emphasizing the need to strengthen key core technologies and improve the innovation capabilities of the industry. Promote digitalization, networking and intelligence in manufacturing, and strengthen the application of energy-saving and environmental protection technologies, processes and equipment. The event also calls for the full implementation of efficient, clean, low-carbon, recyclable green manufacturing systems.

The high-tech industry (HTI), with characteristics of intensive knowledge and technology, high investment of R&D activity and R&D personnel, low energy consumption, and high value added, is one of the keys to promoting and diffusing technological innovation [12]. High-tech industry refers to a collection of enterprises engaged in research, development, production and technical services of one or more high technology and its products. The key technologies possessed by such industries are often difficult to develop, but once developed successfully, they have higher economic contributions and social benefits. The high-tech industry mainly includes three major fields: information technology, biotechnology and new materials technology. According to the “Notice on the Classification of High-tech Industry Statistics” issued by the National Bureau of Statistics of China in July 2002, the statistical scope of China’s high-tech industry includes aerospace aircraft manufacturing, electronics and communication equipment manufacturing, electronic computer and office equipment manufacturing, Pharmaceutical manufacturing and medical equipment and instrumentation manufacturing, etc. In recent years, technological innovation of high-tech industries, such as 5G, artificial intelligence, intelligent terminal, clean energy, high quality graphene production technology etc., promotes the adjustment of regional industrial structure and the rapid development of regional economy in China. Meanwhile, through environmental laws and regulations, tax reduction, issuing pollutant discharge permits, and establishing emissions trading system, the Chinese government actively controls and guides the total pollution discharge of high-tech industries and strengthens environmental regulation of high-tech industries. Nowadays, high-tech industry is playing an important role in achieving green growth in China.

In China, with the innovation-driven development strategy and the implementation of the Belt and Road (B&R) initiative, HTIs in the provinces (municipalities) along the B&R are promoting the transformation of regional economic development patterns along the B&R, which are becoming important driving forces in China for solving structural imbalances and maintaining green growth.

**Figure 1:**
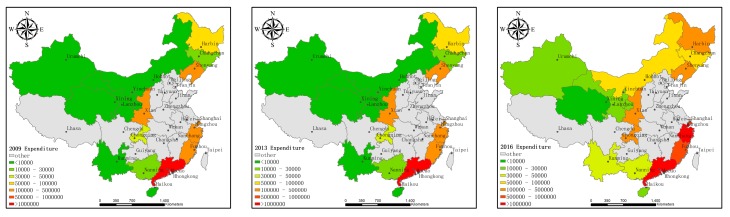
Intramural Expenditure on R&D in high-tech industries (excluding Xizang) in the provinces (municipalities) along the Belt and Road (B&R) in 2009, 2013, and 2016.

**Figure 2:**
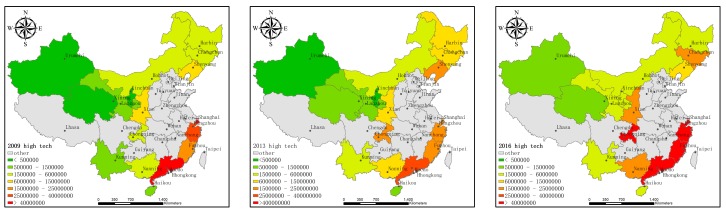
Main business income of high-tech industries (excluding Xizang) in the provinces (municipalities) along the B&R in 2009, 2013, and 2016.

**Figure 3:**
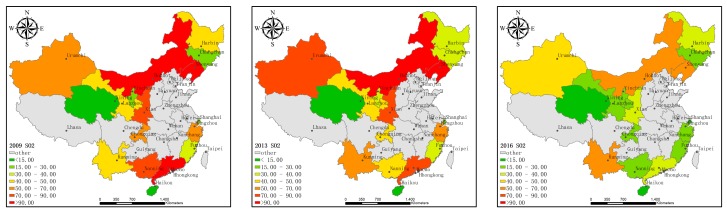
Sulfur dioxide emissions from high-tech industries (excluding Xizang) in the provinces (municipalities) along the B&R in 2009, 2013, and 2016.

Figure 1, Figure 2 and Figure 3 show the technological innovation input, the economic output, and pollutant emissions of HTI in each province (municipality) along the B&R in 2009, 2013, and 2016, represented by the intramural expenditure on R&D, main business income, and sulfur dioxide emissions, which give an aerial view about the change and trend of HTI along the B&R in these years.

There are 18 provinces (municipalities) along the B&R in China, including Shanghai, Fujian, Guangdong, Zhejiang, and Hainan along the Maritime Silk Road and Chongqing, Shanxi, Gansu, Ningxia, Qinghai, Neimenggu, Heilongjiang, Jilin, Liaoning, Guangxi, Xinjiang, Xizang, and Yunnan along the Silk Road Economic Belt. Xizang is not shown in Figure 1, Figure 2 and Figure 3 because of the lack of data. The data comes from the 2009, 2013, and 2016 China High-Tech Industry Statistical Yearbooks.

Figure 1 shows that the research investment in the HTI before 2013 has not changed significantly. Compared with 2013, the investment in scientific research in 2016 increased significantly. Figure 2 shows that, in terms of economic output, the income of the HTI in 2013 increased marginally compared to 2009. The overall increase in 2016 was significantly higher than in 2013. As seen in Figure 3, the pollutant emissions from the HTI along China’s Maritime Silk Road decreased from 2009 to 2013 and the overall pollutant emissions decreased significantly in 2016 than in 2013. Overall, these three sets of maps show the general trend of the development of HTI along the B&R in China. Since the launch of the B&R initiative in 2013, high-tech industries along the B&R in China have increased their investment in technological innovation and economic prosperity and the emission of environmental pollutants has decreased. This shows that, in recent years, HTI along the B&R in China seems to be committed to achieving green growth. Therefore, this study proposed to measure the efficiency of the innovation activities of the high-tech industry to further study the decomposition of the specific changes, as well as their main influencing factors, in order to rationally and effectively use innovative resources, promote the regional economy, and coordinate the environment.

This paper is divided as follows: Section 2 reviews the studies on the technological innovation efficiency of the high-tech industry with different aspects, methods, and perspectives. Section 3 provides the model, method, and data for this study. Section 4 discusses the results. Section 5 details conclusions, policy implications, and limitations.

## 2. Literature Review

The role of HTI in technological progress and economic growth has become increasingly prominent in recent years. Research on technological innovation efficiency (TIE) has always been a hot topic at home and abroad. To date, a large number of studies have focused on different aspects, methods, and perspectives:

1. Some scholars have studied the TIE among different high-tech industries from the perspective of industry differences [13,14,15], while many others study the TIE of HTI from the perspective of regional differences [16] or compare the TIE of HTI at a provincial level in China [17,18], or focus on a particular province or city [19,20,21,22]. In recent years, however, scholars have paid more attention to the development of regional economies, research on the TIE of HTI in a certain economic zone has increased than ever before, especially for the Yangtze River economic belt and the Silk Road Economic Belt, and research on the TIE of regional HTI has increased [23,24,25].
2. From the perspective of research content, scholars treated HTI’s innovation activities as black boxes in the past and the internal mechanism and transformation process of the HTI innovation system has not been clear. In recent years, with the increased understanding of the innovation process and value chain of HTI, scholars have begun to decompose the efficiency of different stages of technological innovation from the value chain perspective [26,27,28,29,30]. In addition, with the extensive attention of the international community on ecological and environmental issues, the environmental impact of technological innovation on enterprise has gradually received attention [31]. Compared with traditional technological innovation, enterprise should achieve sustainable development of technology, environment, and economy by saving energy and reducing pollutant emissions. Therefore, more scholars have begun to explore the environmental, economic, and social integration of sustainable TIE [32,33,34,35].
3. From the perspective of research methods, parametric and nonparametric research methods are used in the study of TIE in HTI. Among them, the parametric method is based on the stochastic frontier analysis (SFA), but there is a deviation in the function model setting. The nonparametric method is based on data envelopment analysis (DEA). Some scholars suggest the two-stage DEA method to measure industrial TIE and its influencing factors, which makes up for the defects of the traditional DEA model [36,37]. The two-stage DEA model can analyze the influencing factors of TIE but still cannot eliminate the influences of environmental factors and random errors. Therefore, the three-stage DEA model, which eliminates the influences of environmental factors and random errors with the help of the SFA model, is increasingly being used by scholars [38]. However, the technological innovation process often leads to a loss of expected output (undesirable output), and ignoring this would significantly influence the innovation efficiency value. A small number of scholars consider the unexpected output when studying TIE in HTI [39]. Most of the above are static efficiency analyses, which cannot accurately reflect the dynamic change in TIE. Some scholars use the DEA–Malmquist model to decompose the change of TIE into technical efficiency change and technical progress and analyze the changing trends of TIE in HTI [40,41].

To sum up, the research on the TIE of the HTI in the above literature has the following deficiencies: (1) the research results of the TIE of HTI are constantly enriched, and most of them are limited to exploring TIE from the social economy perspective and analyzing the internal process of TIE. However, from the sustainable development perspective, HTI needs to change the previous development mode; therefore, it must put environmental problems on the same level as technological progress and economic progress and consider the environmental pollution problems brought about by technological innovation, in order to calculate and evaluate the TIE more comprehensively and accurately. (2) Most studies only carry out a static efficiency analysis when considering no expected output; this cannot accurately reflect the dynamic change of TIE. For research on the dynamic change of TIE, however, most scholars use the Malmquist–Luenberger (ML) index for TIE and its decomposition, but this does not meet the requirements of transitivity in form. When calculating the intertemporal directional distance function, there are no feasible solutions such as linear programming and it is difficult to compare the efficiency across time-series. (3) Most of the research on TIE is at the national level, and there is a lack of in-depth research on HTI along the B&R. Under the background of an innovation-driven development strategy and the B&R initiative, and based on the coordinated and sustainable development of technology, environment, and economy, it is more realistic to study the changing trends and influencing factors of HTI’s innovation efficiency along the B&R, which also has more practical guidance and reference value.

Considering the existing research limitations described above, this study selected HTI along the B&R in China as the research object from a sustainable development perspective, considers the undesirable output, comparatively analyzes the TIE at the provincial level, discusses the efficiency within the time sequence characteristics of dynamic change, and finally discusses the influencing factors. The global Malmquist–Luenberger (GML) index can better solve the above defects of the ML index, and the continuous global production frontier can avoid technological regression and the occurrence of passive productivity improvement [42]. It can not only reflect the dynamic change of efficiency but can also make an intertemporal comparison of efficiency.

After comprehensive consideration and based on the directional distance function and global Malmquist—Luenberger index model (DDF–GML), this study used the MaxDEA Ultra 8 software to measure the HTI’s TIE and its dynamic changes, with environmental pollution as the undesirable output. Based on the natural fracture clustering analysis method, ArcGIS 10.2 software was used to characterize the spatial distribution of TIE in HTI along the B&R. Finally, based on the panel Tobit model, Stata 14.0 software was used to calculate the influencing factors.

## 3. Model, Variables, and Data

### 3.1. Model

#### 3.1.1. Directional Distance Function Model Considering Undesirable Output

This study incorporated undesirable outputs into the input—2014output efficiency evaluation, which not only improves the effectiveness of the efficiency measurement but is also closer to the needs of sustainable development. First, this study constructed a set of production possibilities that included both expected output (or “good” output) and undesirable output (or “bad” output). This study took each province (municipality) along the B&R in China as a decision-making unit, assuming that each decision-making unit uses N items of input ($x=x_{1}\;,\ldots ,x_{n}\;\in R_{N}^{+}$), K items of expected output ($y\;=y_{1}\;,\ldots ,\;y_{k}\;\in \;R_{K}^{+}$), and J items of undesirable output ($b=b_{1}\;,\ldots ,b_{j}\in {\;R}_{J}^{+}$). X represents the inputs of technological innovation activities, y represents expected outputs, b represents undesirable outputs. Then, the current production possibility set can be constructed by the input—output combination of N decision units, which can be expressed as:

(1) $$P^{t}\left(x^{t}\right)=\left\{\left(y^{t},b^{t}\right){|x}^{t}\;\mathrm{produces}\;\left(y^{t},b^{t}\right)\right\},\;t=1,\;\cdots ,\;T$$

Equation (1) indicates that input x can produce expected output y and undesirable output b and needs to satisfy the following assumptions: (1) no free lunch—under the condition that the output of undesirable output is zero, expectation output will not be produced, (2) boundedness—under certain conditions, expected output and undesirable output are limited, (3) strongly disposable—inputs and expected outputs can be obtained at any production point below the production front, (4) weakly disposable—the reduction of undesirable output is costly, and (5) convexity—production technology conforms to the law of diminishing marginal production [43].

Chung et al. argued that the directional distance function method can solve the problem of efficiency evaluation involving undesirable outputs [43]. Let the directional vector be $g=\left(g_{y},g_{b}\right)$, $g\in R_{K}^{+}\times R_{J}^{+}$, β is the value of the directional distance function that maximizes the expected output and minimizes the undesired output, *P(x)* is the current period production possibility set. The corresponding DDF is defined as:

(2) $$D\left(x,y,b;g_{y},g_{b}\right)=max\left\{\beta |(y+\beta g_{y},\;b-\beta g_{b}\right\}\in P\left(x\right)$$

#### 3.1.2. Global Malmquist—Luenberger Index Method

In order to overcome the two drawbacks—the traditional ML index is not cyclical and there is no feasible solution in linear programming—Oh (2010) built a common frontier based on the improvement of the traditional ML index from a global perspective, analyzed the change of total factor productivity, and proposed the GML index [44]. He introduced two concepts—current production possibility set and global production possibility set. The current production possibility set is expressed as Equation (2), and the global production possibility set represents the union of all the current production feasible sets, that is, $P^{G}=P^{1}\cup P^{2}\cup \cdots P^{T}$; this collection also covers the current set of production possibilities for all observed units.

The GML productivity index from the t to the t+1 period and its decomposed form are expressed as follows:

(3) $${\mathrm{GML}}^{t,t+1}\left(x^{t},y^{t},b^{t},x^{t+1},y^{t+1},b^{t+1}\right)=\frac{1+D^{G}\left(x^{t},y^{t},b^{t}\right)}{1+D^{G}\left(x^{t+1},y^{t+1},b^{t+1}\right)}\;\begin{array}{l} =\frac{1+D^{t}\left(x^{t},y^{t},b^{t}\right)}{1+D^{t}\left(x^{t+1},y^{t+1},b^{t+1}\right)}\times \left[\frac{\left(1+D^{G}\left(x^{t},y^{t},b^{t}\right)\right)/\left(1+D^{t}\left(x^{t},y^{t},b^{t}\right)\right)}{\left(1+D^{G}\left(x^{t+1},y^{t+1},b^{t+1}\right)\right)/(1+D^{t+1}\left(x^{t+1},y^{t+1},b^{t+1}\right)}\right]\\ ={\mathrm{GMLEC}}_{t}^{t+1}\times {\mathrm{GMLTC}}_{t}^{t+1} \end{array}$$

In Equation (3), $D^{G}\left(x,y,b\right)=max\left\{\beta |(y+\beta y,\;b-\beta b\right\}\in P^{G}\left(x\right)$ is the global directional distance function, which depends on the global production possibility set $P^{G}\left(x\right)$. $GMLEC^{t,t+1}$ represents the changes in technical efficiency during two time periods, and $GMLTC^{t,t+1}$ represents technological progress during the two time periods.

In order to measure and decompose the GML index, we used the DEA linear programming model to solve the four directional distance functions in equation 3. Taking the t period as an example, the current directional distance function $D^{t}\left(x^{t},y^{t},b^{t}\right)$ and the global directional distance function established on the global production possibility set $D^{G}\left(x^{t},y^{t},b^{t}\right)$ can be obtained by solving the following linear programming, respectively:

(4) $$D^{t}\left(x^{t},y^{t},b^{t}\right)=max\beta \;s.t.Y^{t}z^{t}\geq \left(1+\beta \right)y_{k}^{t}\;B^{t}z^{t}=\left(1-\beta \right)b_{k}^{t}\;X^{t}z^{t}\leq x_{k}^{t}\;z^{t}\geq 0$$

(5) $$D^{G}\left(x^{t},y^{t},b^{t}\right)=max\beta \;s.t.\overset{T}{\underset{\tau =1}{\sum }}Y^{\tau }z^{\tau }\geq \left(1+\beta \right)y_{k}^{t}\;\overset{T}{\underset{\tau =1}{\sum }}B^{\tau }z^{\tau }=\left(1-\beta \right)b_{k}^{t}\;\overset{T}{\underset{\tau =1}{\sum }}X^{\tau }z^{\tau }\leq x_{k}^{t}\;z^{\tau }\geq 0$$

Similarly, the current directional distance function of the t+1 period can be obtained by $D^{t+1}\left(x^{t+1},y^{t+1},b^{t+1}\right)$ and the global direction distance function by $D^{G}\left(x^{t+1},y^{t+1},b^{t+1}\right)$.

#### 3.1.3. Panel Data Tobit Regression Model

Since the efficiency value measured by the DDF—GML model is greater than 0, the data are truncated. Using the ordinary least squares method will lead to bias and inconsistency. Therefore, the panel data Tobit regression model was used in this study to analyze the influencing factors of TIE [45,46,47]:

(6) $$\{\begin{array}{c} Y_{i}^{*}=\beta^{T}x_{i}+\varepsilon_{i}\\ Y_{i}=Y_{i}^{*},\;Y_{i}^{*}>0\\ Y_{i}=0,\;Y_{i}^{*}\leq 0 \end{array}\varepsilon_{i}\sim N\left(0,\sigma^{2}\right),\;i=1,2,3,\cdots ,n$$

In the equation above, $Y_{i}^{*}$ is an independent variable, $x_{i}$ is a dependent variable, and $\beta^{T}$ is the correlation index.

### 3.2. Variables

#### 3.2.1. Input and Output Variables

Technology innovation in high-tech industries is a dynamic process of continuous multi-input and multi-output development and change. The indicators needed to measure TIE include input and output variables (Table 1). This study drew on the research results of scholars in the selection of input and output indicators for technological innovation in HTI [48,49,50,51]. R&D personnel for full-time equivalents and R&D personnel were carefully selected from the labor input perspective. From the capital investment perspective, R&D intramural expenditure and new product development funds were selected as input indicators. The number of patent applications, number of new product development projects, and the sales revenue of new products were selected as output indicators from the intermediate output and final output perspective, reflecting the technological innovation achievements of HTI. The industry pollutant emission often considers include industrial sulfur dioxide emissions, industrial nitrogen oxide emissions, and industrial smoke (powder) dust emissions, the trend of which are same during study period, so the sulfur dioxide emissions of HTI were selected as indicator of undesirable output from the perspective of environmental pollution emissions in the process of technological innovation.

#### 3.2.2. Influencing Factors

Many factors affect TIE in China’s provincial industrial enterprises. Based on previous studies, five control variables were taken into consideration: government support (GOV), economic openness (OPE), R&D intensity (RD), laborer’s quality (LAB), and environmental regulation (ER). This study focused on the impact of these factors on the TIE of HTI. Among them, GOV uses the proportion of government investment in the internal expenditure of R&D funds, LAB is represented by the proportion of scientific and technological researchers in the industry, OPE represents the HTI import and export trade in GDP, RD is the proportion of the internal expenses of R&D funds in the main business income, and ER is the proportion of industrial environmental pollution control in GDP [52,53,54]. The relevant metrics and their codes are shown in Table 2.

### 3.3. Data Source and Processing

**Figure 4:**
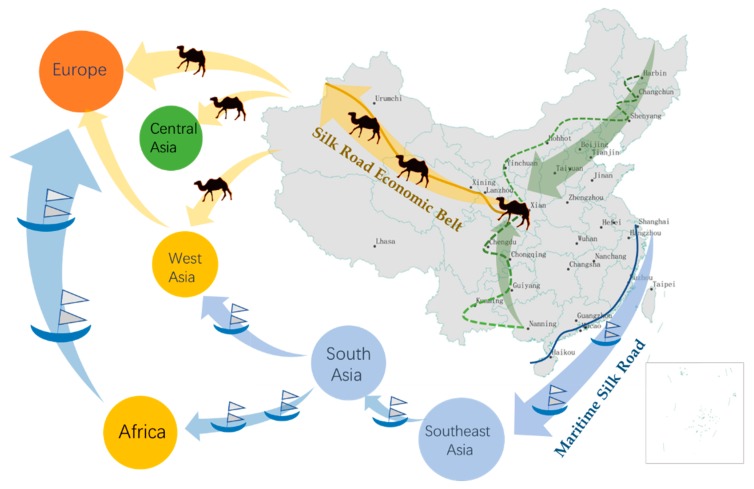
Provinces (municipalities) along the B&R (Shanghai, Fujian, Guangdong, Zhejiang, and Hainan along the Maritime Silk Road and Chongqing, Shanxi, Gansu, Ningxia, Qinghai, Neimenggu, Heilongjiang, Jilin, Liaoning, Guangxi, and Yunnan along the Silk Road Economic Belt).

This study took the HTI of 16 provinces (municipalities) along the B&R in China as the research object, including Shanghai, Fujian, Guangdong, Zhejiang, and Hainan along the Maritime Silk Road and Chongqing, Shanxi, Gansu, Ningxia, Qinghai, Neimenggu, Heilongjiang, Jilin, Liaoning, Guangxi, and Yunnan along the Silk Road Economic Belt, as shown in Figure 4. Owing to missing data, Xinjiang and Xizang were not included. The research period ranged from 2009 to 2016, eight years in total. I3, I4, and O3 were converted to constant prices in 2000 with the GDP deflator, after considering inflation.

The data on the desirable outputs and the inputs were collected from the China Statistical Yearbook on High Technology Industry (2010–2017) and the China Statistical Yearbook on Science and Technology (2010–2017). The data on the industrial SO_2_ emissions were obtained from the China Statistical Yearbook on Environment (2010–2017). The data on the GDP were derived from the China Statistical Yearbook (2010–2017). The data on the influencing factors were obtained from the China Statistical Yearbook on High Technology Industry and the China Ecological Statistical Yearbook (2010–2017) [55,56,57,58,59,60,61,62].

## 4. Results and Discussion

### 4.1. Technological Innovation Efficiency

Based on the DDF—GML index model with the undesirable outputs (as mentioned above), and taking the HTI of 16 provinces (municipalities) along the B&R as the research object, the TIE from 2009 to 2016 was calculated and decomposed using MaxDEA Ultra 8 software (Beijing Realworld Software Company Ltd., Beijing, China). The results are shown in Table 3. GML, EC, and TC are the input-output efficiency, technical efficiency change, and technological progress of the decision-making unit respectively; GML, EC, and TC are greater than (less than) 1, respectively indicating that the input-output efficiency level is increased (lower), the technical efficiency is increased (decreased), the technological progress (regression). By analyzing the GML index and its decomposition items, we can observe the trend of the efficiency of technological innovation activities and the changes of influencing factors.

Table 3 shows that, when considering undesirable output, the TIE of the HTI in all provinces (municipalities) along the B&R between 2009 and 2016 increased. The cumulative change value and geometric mean of the GML index were 1.0941 and 1.0101, respectively, which indicate that the overall growth rate of green technology innovation was 9.41% under environmental constraints; correspondingly, the average annual growth rate was 1.01%. From the decomposition of the GML index, the cumulative change value and geometric mean of technical efficiency were less than 1, and the average change rate was −4.22% and −0.45%, respectively, indicating that the overall technical efficiency was slightly down. The cumulative growth rate of technological progress was 12.69%, with an average annual growth rate of 1.48%, technological progress was characterized by rapid growth. Therefore, although the technical efficiency of HTI declined every year on average, the technological progress was increased, so it still showed an increasing trend in the input-output efficiency of whole technological innovation activities. This indicates that the improvement of TIE in HTI in the provinces (municipalities) along the B&R is mainly dependent on the promotion of technological progress and that the decline in technological efficiency has inhibited the growth of TIE.

**Figure 5:**
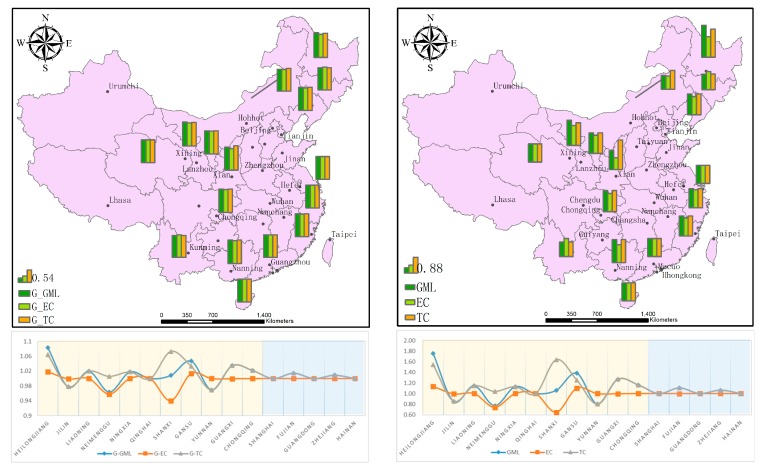
The 2009–2016 GML Index of TIE in HTI for provinces (municipalities) along the B&R in China and its decomposition. (**a**) The geometric mean of GML(G-GML), EC(G-EC) and TC(G-TC). (**b**) The cumulative change value of GML, EC and TC.

As can be seen from Table 3 and Figure 5, between 2009 and 2016, under environmental constraints, the cumulative growth rate of the GML index in HTI along the Silk Road Economic Belt and the Maritime Silk Road Economic Belt was 12.13% and 3.44%, respectively, corresponding to an average annual growth rate of 1.25% and 0.47%, respectively. It can be seen that the growth rate of the TIE of the Silk Road Economic Belt was significantly higher than that of the Maritime Silk Road. As far as the decomposed GML index is concerned, the cumulative change value and geometric mean of the technical efficiency of the Silk Road Economic Belt were less than 1 and the average rate of change was −4.64% and −0.65%, respectively, while the technical efficiency of the Maritime Silk Road remained unchanged (on average). The accumulated value and geometric mean of technological progress in the HTI of the Silk Road Economic Belt increased by 16.88% and 1.94%, respectively, which were significantly higher than that of the Maritime Silk Road. The fundamental reason for the improvement of the TIE of HTI in the Silk Road Economic Belt is technological progress, and the decline in technological efficiency has hindered the substantial improvement of TIE. Although the TIE of HTI on the Maritime Silk Road has not improved greatly, the result is because of continuous technological progress while maintaining the same technical efficiency.

According to the specific calculation of the GML index and its decomposition in various provinces and municipalities, the cumulative growth rates of the GML index in Heilongjiang, Liaoning, Ningxia, Gansu, Guangxi, Chongqing, and Fujian were all over 10%. The HTI in both Heilongjiang Province and Gansu Province has improved in technological efficiency and technological progress. The cumulative increase of TIE in Heilongjiang Province was as high as 75.35%, the cumulative increase of technological efficiency was 15.55%, and the cumulative increase of technological progress was 54.43%. Similarly, Gansu Province not only had improved technological progress but also technical efficiency. Although TIE improved in Guangxi and Shaanxi, according to the decomposition of the GML index, technological progress increased by 63.83% and 27.29%, respectively, while technological efficiency declined by −35.22% and −0.34%, respectively. The TIE of high-tech enterprises in Shaanxi Province showed an increasing trend as a whole, because the growth of technological progress was higher than the decline of technological efficiency by a large margin. The inefficiency of input—output and the high growth of pure technological progress illustrate that Shaanxi Province achieved higher growth in pure technological progress by strengthening the promotion of technological innovation at a higher speed and introducing a large number of talents and investments, while the output in the process of technological innovation was time-lagging and showed a lower input–output efficiency in the short term. This is in line with the actual development of HTI in Shaanxi Province. The cumulative values of the GML index in Jilin, Neimenggu, and Yunnan were all less than 1, which shows that the TIE in these three provinces had decreased. Although Neimenggu showed technological progress, its technological efficiency had decreased. Jilin and Yunnan showed a double decline in technological progress and technological efficiency.

In order to further compare the differences in the TIE between the provinces and municipalities along the provincial road, the variation trend and the power source of the TIE are discussed. Table 4 shows the annual variation of the GML index and its decomposition value of TIE in HTI along the B&R from 2009 to 2016.

**Figure 6:**
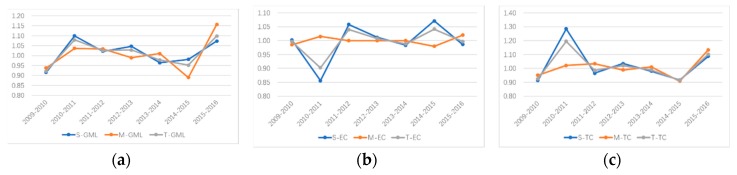
The annual variation of the GML index and its decomposition value of TIE from 2009 to 2016. (**a**) represents GML value of Silk Road Economic Belt (S-GML), Maritime Silk Road(M-GML) and Belt and Road(T-GML); (**b**) represents EC value of Silk Road Economic Belt (S-EC), Maritime Silk Road(M-EC) and Belt and Road(T-EC); (**c**) represents TC value of Silk Road Economic Belt (S-TC), Maritime Silk Road(M-TC) and Belt and Road(T-TC).

As can be seen from Table 4 and Figure 6a, the GML index reflecting the change in TIE shows that, from 2009 to 2016, the TIE of HTI in all provinces (municipalities) along the B&R line presents an overall trend of fluctuation and increase, decreasing yearly from 2013 to 2015 and then increasing significantly from 2015 to 2016. After decomposing the GML index, by comparing Figure 6b,c, it can be observed that the technical efficiency of HTI along the Silk Road Economic Belt increased after a sharp decline from 2010 to 2012 and then continued to decline. Technological progress rose sharply and then fell again, which shows that despite the improvement in technical efficiency during this period the HTI of the Silk Road Economic Belt witnessed extensive development because of the unreasonable allocation of input and output resources.

Despite technological progress, resources are wasted, and this pollutes the environment. The technical efficiency of HTI along the Maritime Silk Road fluctuated slightly before 2014. Technological efficiency and technological progress both declined from 2014 to 2015 and then significantly increased from 2015 to 2016. This shows that under the One Belt and One Road initiative, the HTI of the Maritime Silk Road has increased investment and R&D and has actively adapted and adjusted the mode of technological innovation; technological efficiency and technological progress greatly improved only in 2016. Similarly, HTI in both the Silk Road Economic Belt and the Maritime Silk Road Economic Belt tended to have the same changes in TIE and technological progress from 2014 onwards. In addition, technical efficiency improved between 2014 and 2015. Although it declined between 2015 and 2016 because of the advantages of technological progress, the overall TIE improved.

### 4.2. Analysis of Influencing Factors

This study used the data of the provincial and municipal high-tech industrial TIE along the B&R from 2009 to 2016 for empirical analysis. The sample data had a short duration and many cross-sectional units—which were typically short-panel data—and individual differences between the units were considered. Therefore, the panel random Tobit model for empirical analysis was selected for this study. Combining the characteristics of provincial HTI along the B&R, five basic factors were selected to analyze the changes in the TIE of HTI and the following Tobit regression model was constructed:

(7) $$GMLE_{i}=\beta_{0}+\beta_{1}GOV_{i}+\beta_{2}LAB_{i}+\beta_{3}OPE_{i}+\beta_{4}RD_{i}+\beta_{5}ER_{i}+\varepsilon_{i}$$

In the above equation, GMLE is the global technical innovation efficiency; *β*_0_ is the intercept term; *β*_1_, *β*_2_, *β*_3_, *β*_4_, and *β*_5_ are the regression coefficients of their respective variables; i represents the period (i = 1, 2, 3, and 4), and ε is the residual term. There are five factors in the formula: government support (GOV), economic openness (OPE), R&D intensity (RD), laborer’s quality (LAB), and environmental regulation (ER). This study focused on the impact of these factors on the TIE of HTI. Among them, GOV uses the proportion of government investment in the internal expenditure of R&D funds, LAB is represented by the proportion of scientific and technological researchers in the industry, OPE represents high-tech industry import and export trade in GDP, RD is the proportion of the internal expenses of R&D funds in the main business income, and ER is the proportion of industrial environmental pollution control in GDP. Using the above constructed model and using Stata 14.0 software for regression, the following results were obtained, as shown in Table 5.

As can be seen from Table 5, both the Wald—χ2 value and the Likelihood Ration test (LR test) value were significant at the 1% level, which indicates that the model has a good fit. When considering individual effects, panel random Tobit regression should be used. The impact of most of the explanatory variables on the TIE was consistent with the relevant analysis in other research or economic disciplines or in line with the development of the B&R region. This shows that the equation has a strong explanatory power for the relationship between variables and that the regression results are reliable. Based on the empirical analysis results, we can understand the following:

1. The impact of government financial support on the TIE is not significant. The government should play an auxiliary role, providing better policies and mechanisms to guide the technological innovation direction of HTI, rather than participating and investing in technological innovation and R&D. Companies with stronger R&D capabilities and greater demand for technological innovation are willing to invest more in R&D of new technologies and products rather than relying on government and financial institutions;
2. The elasticity coefficient of R&D intensity is negative for the TIE and is significant at the 95% confidence level. This shows that the intensity of R&D has a significantly negative impact on the TIE. When the R&D intensity is too high, too much investment is likely to cause the waste of R&D resources. R&D results cannot be quickly converted into economic benefits; this may lead to an increase in technological innovation but too much of this will lead to a lower TIE. During the research period, it was observed that most industries were at a stage of higher technological progress and lower technical efficiency, and therefore, even when the R&D investment is increased, TIE cannot be improved. On the contrary, this may lead to the waste of investment in R&D resources, which could further lead to a decline in technical efficiency;
3. The elasticity coefficient of the quality of laborers to the TIE is positive, and it is significant at the 95% confidence level, which indicates that the positive correlation between the quality of laborers and the TIE of HTI is significant. The higher the quality of R&D personnel in HTI, the easier it is to improve the TIE. Therefore, HTI should attach importance to and strengthen the introduction of talent;
4. The degree of openness to the outside world has a significant positive impact on the TIE. Openness can not only bring in funds but also introduces technology. In particular, the introduction of technology in HTI can significantly increase green technology innovation capabilities. The provinces and municipalities along the B&R have benefited from the “One Belt, One Road” strategy. Foreign trade can rapidly introduce advanced science and technology and management methods, with obvious advantages for technological innovation. Therefore, foreign trade can significantly promote the rise of TIE;
5. The elasticity coefficient of environmental regulation on the TIE is positive and significant at the 99% confidence level. This shows that environmental regulation has a significant positive impact on the TIE. Reasonable and rigorous environmental regulation can promote technological innovation activities, in line with the Potter effect in economics. The government’s environmental regulation can guide HTI to enhance technological innovation while considering the constraints of environmental pollution emissions. The more effective environmental regulations are, the higher the TIE, which considers undesirable environmental outputs.

## 5. Conclusions, Policy Implications, and Limitations

### 5.1. Main Conclusions

Based on the data of 16 provinces (municipalities) along the B&R in China, this study used the DDF—GML index model to measure the HTI’s TIE and its dynamic changes, while considering environmental pollution as the undesirable output. Through the measurement and decomposition of TIE, from the geography and time-series dimensions, the dynamic changes in the TIE of the provincial HTI along the B&R during the study period were elaborated and compared, which was intuitively and clearly expressed using ArcGIS 10.5 software. On this basis, this study used the panel data Tobit model to empirically study the influencing factors of TIE. The main findings are as follows:

1. In general, between 2009 and 2016, with environmental immersion as the undesirable output, the TIE of the HTI along the B&R fluctuated, however, it showed a rising trend. The overall TIE showed that the HTI along the B&R had not yet achieved green growth. The results of the TIE decomposition show that the increase in TIE was mainly brought about by technological progress and that the decline in technical efficiency hindered the improvement of TIE. This indicates that during the study period, HTI along the B&R generally attached importance to R&D and investment in technological innovation, ignoring the problem of input and output resource allocation, which resulted in excessive R&D capital investment and waste of R&D resources. Therefore, HTI should actively adjust the innovation structure and model, attach importance to the waste of scientific research resources, and accelerate the commercialization and marketization of technological achievements;
2. From the perspective of geography, the TIE of HTI along the Silk Road Economic Belt from 2009 to 2016 was higher than that of the Maritime Silk Road. This improvement was brought about by technological advancement and an inefficient allocation of resources. The utilization rate of innovation resources was low, and the cost of resource consumption and environmental pollutant emissions was higher than economic benefits. However, combined with the results of time-series data, the TIE of HTI along the Silk Road Economic Belt and the Maritime Silk Road Economic Belt also fell sharply, with the turning point between 2014 and 2015. However, it rose sharply in 2015–2016, and this fluctuation was mainly because of technological progress. This shows that high-tech enterprises were affected by the promotion and implementation of the B&R initiative and the innovation-driven strategy proposed by China in 2013–2014 and that the development mode of technological innovation was constantly adjusted. The improvement in TIE during 2015–2016 was high, which shows that the technology industry successfully adjusted the technological innovation and economic development model and began to shift to the sustainable green development of TIE, environment, and economy;
3. Considering the influencing factors, the results from the 2009–2016 study period show that the government’s direct funding support had no impact on the improvement of technological innovation in HTI. On the contrary, the government’s regulation of environmental pollution can significantly affect the efficiency of industrial technology innovation. The higher the level of R&D personnel in HTI, the more helpful it is to improve the TIE. Therefore, HTI should pay attention to cultivating and introducing high-tech talents. Additionally, TIE can be promoted by strengthening cooperation with other countries along the B&R and increasing the amount of foreign trade in HTI.

### 5.2. Policy Implications

Between 2009 and 2016, HTI along the B&R initiative did not achieve green growth. However, a trend toward green sustainable development could be observed. Although the TIE in HTI improved during the research period, there was still too much emphasis on R&D investment, while ignoring technical efficiency. The government, as an auxiliary institution rather than a R&D participant, should assist with guidance, regulation, and support. It should allow enterprises to become the main body for technology demand selection and technical project determination, and consequently, the main body for technological innovation investment and the industrialization of innovation results. The government can provide talent introduction, training policies, and environmental pollution control mechanisms, along with providing opportunities for cooperation between Chinese high-tech enterprises and foreign companies. This is more conducive to cultivating high-tech achievements and talent in HTI, enhancing their own technological innovation strength, and paying attention to the rational allocation of innovative resources. We can ensure the sustainable development of HTI only by reducing consumption, reducing pollution, and changing the development model of the excessive consumption of resources and environmental pollution.

### 5.3. Limitations

Although this study presents valuable theoretical and practical implications, some limitations must be addressed. Firstly, considering the same growth trends of other pollutants emissions (such as CO_2_, industrial NOx emissions, and industrial smoke & powder dust emissions) throughout the study period, this paper only used SO_2_ as the undesirable output to represent the environment pollutant emission. Further research could include more emissions as environmental control variables. Including more pollutants as the control variables may lead results changes and puts more weight on environmental degradation on technological innovation of high-tech industry, further research should also discuss the weight of each pollutant variables. Secondly, due to the lack of data in some provinces in certain years, this study period was less than 10 years, only from 2009–2016, so it only showed that high-tech industry along the B&R in China is on the way to sustainable green growth. It’s highly possible that the growth rate will be faster than expected with the B&R initiative and the support by Chinese government in future. Therefore, future research can consider longer study period, for example from 2013–2023, and continue to focus on the field of sustainable green growth of high-tech industry along B&R in China.

## Figures and Tables

**Table 1:** Input and output variables of technological innovation efficiency (TIE) in high-tech industry (HTI) along the B&R.

Index	Variable Abbreviation	Measurement Method	Unit
Input	I1	Full-time Equivalent	man-year
	I2	R&D Personnel	10,000 yuans
	I3	Intramural Expenditure on R&D	10,000 people
	I4	Expenditure on New Product Development	10,000 yuans
Expected Output	O1	Patent Applications	piece
	O2	New Products	item
	O3	Sales Revenue of New Products	10,000 yuans
Undesirable Output	O4	Volume of Sulfur Dioxide Emission by Industry	1000 ton

**Table 2:** Influencing factors of TIE in HTI along the B&R.

Variable Abbreviation	Variables	Measurement Method	Unit
GOV	Government Support	The funding of R&D comes from the government	10,000 yuan
OPE	Economic openness degree	Import and export trade volume	100 million yuan
RD	R&D intensity	The ratio of internal R&D expenditure to the main business income	%
LAB	Laborer’s Quality	The ratio of internal R&D expenditure to the main business income	%
ER	Environmental Regulation	Pollution control accounts for the proportion of GDP	%

**Table 3:** The 2009–2016 global Malmquist—Luenberger (GML) Index of the TIE of HTI in Provinces (municipalities) along the B&R in China and its Decomposition.

Region	Cumulative Change Value			Geometric Mean		
	GML	EC	TC	GML	EC	TC
Heilongjiang	1.7535	1.1355	1.5443	1.0835	1.0183	1.0641
Jilin	0.8516	0.9930	0.8576	0.9773	0.9990	0.9783
Liaoning	1.1462	1.0001	1.1462	1.0197	1.0000	1.0197
Neimenggu	0.7719	0.7404	1.0426	0.9637	0.9580	1.0060
Ningxia	1.1305	1.0000	1.1305	1.0177	1.0000	1.0177
Qinghai	0.9999	1.0000	0.9999	0.9999	1.0000	0.9999
Shanxi	1.0612	0.6478	1.6383	1.0085	0.9399	1.0731
Gansu	1.3828	1.1002	1.2569	1.0474	1.0137	1.0332
Yunnan	0.8039	0.9995	0.8039	0.9693	0.9999	0.9693
Guangxi	1.2686	0.9966	1.2729	1.0346	0.9995	1.0351
Chongqing	1.1635	1.0000	1.1635	1.0219	1.0000	1.0219
Shanghai	1.0000	1.0000	1.0000	1.0000	1.0000	1.0000
Fujian	1.1070	0.9991	1.1079	1.0146	0.9999	1.0147
Guangdong	1.0000	1.0000	1.0000	1.0000	1.0000	1.0000
Zhejiang	1.0651	1.0000	1.0651	1.0090	1.0000	1.0090
Hainan	1.0000	1.0000	1.0000	1.0000	1.0000	1.0000
Silk Road Economic Belt	1.1213	0.9536	1.1688	1.0125	0.9935	1.0194
Maritime Silk Road	1.0344	0.9998	1.0346	1.0047	1.0000	1.0047
B&R	1.0941	0.9678	1.1269	1.0101	0.9955	1.0148

**Table 4:** The annual variation of the GML index and its decomposition value of TIE from 2009 to 2016.

Period	Silk Road Economic Belt			Maritime Silk Road			Belt and Road		
	GML	EC	TC	GML	EC	TC	GML	EC	TC
2009–2010	0.9150	1.0019	0.9133	0.9374	0.9852	0.9514	0.921936	0.996678	0.925009
2010–2011	1.0991	0.8556	1.2845	1.0365	1.0150	1.0212	1.079127	0.902543	1.195652
2011–2012	1.0215	1.0581	0.9654	1.0335	1.0000	1.0335	1.025215	1.039587	0.986175
2012–2013	1.0467	1.0117	1.0346	0.9894	1.0000	0.9894	1.028431	1.008013	1.020256
2013–2014	0.9639	0.9838	0.9798	1.0101	1.0000	1.0101	0.978104	0.988834	0.989149
2014–2015	0.9811	1.0708	0.9162	0.8901	0.9800	0.9083	0.951709	1.041568	0.913727
2015–2016	1.0728	0.9864	1.0876	1.1570	1.0203	1.1340	1.098406	0.996844	1.101884

**Table 5:** Regression results of factors affecting the efficiency of HTI innovation in provinces (municipalities) along the B&R.

Factors	Coef.	Std. Err.	z	*p* Value
GOV	−0.0747	0.05461	−0.27	0.786
OPE	0.7417 **	0.3779	1.96	0.049
RD	−4.726 **	2.1133	−2.24	0.025
LAB	1.2008 **	0.5971	2.01	0.044
ER	4.53174 ***	1.62105	2.80	0.005
Wald chi2(5) = 15.11 Log likelihood = 47.41 Prob ≥ chi2 = 0.0098 LR test of sigma_u = 0: chibar2(01) = 68.85 Prob ≥ chibar2 = 0.000				

Note: *, **, and *** indicate significant levels at 10%, 5%, and 1%, respectively.
